# Psychometric Properties of the Norwegian Version of the Electronic Health Literacy Scale (eHEALS) Among Patients After Percutaneous Coronary Intervention: Cross-Sectional Validation Study

**DOI:** 10.2196/17312

**Published:** 2020-07-28

**Authors:** Gunhild Brørs, Tore Wentzel-Larsen, Håvard Dalen, Tina B Hansen, Cameron D Norman, Astrid Wahl, Tone M Norekvål, The CONCARD Investigators

**Affiliations:** 1 Clinic of Cardiology St Olavs University Hospital Trondheim Norway; 2 Centre for Child and Adolescent Mental Health, Eastern and Southern Norway Oslo Norway; 3 Norwegian Centre for Violence and Traumatic Stress Studies Oslo Norway; 4 Department of Circulation and Medical Imaging Norwegian University of Science and Technology Trondheim Norway; 5 Department of Internal Medicine Levanger Hospital Nord-Trøndelag Hospital Trust Levanger Norway; 6 Cardiovascular Department Zealand University Hospital Roskilde Denmark; 7 Department of Regional Health Research University of Southern Denmark Odense Denmark; 8 Dalla Lana School of Public Health University of Toronto Toronto, ON Canada; 9 Department of Health Sciences University of Oslo Oslo Norway; 10 Department of Heart Disease Haukeland University Hospital Bergen Norway; 11 Department of Clinical Science University of Bergen Bergen Norway

**Keywords:** eHealth literacy, eHEALS, health literacy, percutaneous coronary intervention, psychometric properties, validation

## Abstract

**Background:**

Web-based technology has recently become an important source for sharing health information with patients after an acute cardiac event. Therefore, consideration of patients’ perceived electronic health (eHealth) literacy skills is crucial for improving the delivery of patient-centered health information.

**Objective:**

The aim of this study was to translate and adapt the eHealth Literacy Scale (eHEALS) to conditions in Norway, and to determine its psychometric properties. More specifically, we set out to determine the reliability (internal consistency, test-retest) and construct validity (structural validity, hypotheses testing, and cross-cultural validity) of the eHEALS in self-report format administered to patients after percutaneous coronary intervention.

**Methods:**

The original English version of the eHEALS was translated into Norwegian following a widely used cross-cultural adaptation process. Internal consistency was calculated using Cronbach α. The intraclass correlation coefficient (ICC) was used to assess the test-retest reliability. Confirmatory factor analysis (CFA) was performed for a priori-specified 1-, 2-, and 3-factor models. Demographic, health-related internet use, health literacy, and health status information was collected to examine correlations with eHEALS scores.

**Results:**

A total of 1695 patients after percutaneous coronary intervention were included in the validation analysis. The mean age was 66 years, and the majority of patients were men (1313, 77.46%). Cronbach α for the eHEALS was >.99. The corresponding Cronbach α for the 2-week retest was .94. The test-retest ICC for eHEALS was 0.605 (95% CI 0.419-0.743, *P*<.001). The CFA showed a modest model fit for the 1- and 2-factor models (root mean square error of approximation>0.06). After modifications in the 3-factor model, all of the goodness-of-fit indices indicated a good fit. There was a weak correlation with age (*r*=–0.206). Between-groups analysis of variance showed a difference according to educational groups and the eHEALS score, with a mean difference ranging from 2.24 (*P*=.002) to 4.61 (*P*<.001), and a higher eHEALS score was found for patients who were employed compared to those who were retired (mean difference 2.31, *P*<.001). The eHEALS score was also higher among patients who reported using the internet to find health information (95% CI –21.40 to –17.21, *P*<.001), and there was a moderate correlation with the patients’ perceived usefulness (*r*=0.587) and importance (*r*=0.574) of using the internet for health information. There were also moderate correlations identified between the eHEALS score and the health literacy domains appraisal of health information (*r*=0.380) and ability to find good health information (*r*=0.561). Weak correlations with the mental health composite score (*r*=0.116) and physical health composite score (*r*=0.116) were identified.

**Conclusions:**

This study provides new information on the psychometric properties of the eHEALS for patients after percutaneous coronary intervention, suggesting a multidimensional rather than unidimensional construct. However, the study also indicated a redundancy of items, indicating the need for further validation studies.

**Trial Registration:**

ClinicalTrials.gov NCT03810612; https://clinicaltrials.gov/ct2/show/NCT03810612

## Introduction

Electronic health (eHealth) delivery provides an opportunity to redesign and improve health care services and health information using web-based technologies that can be accessed over the internet following diagnosis and discharge from hospital [1]. eHealth interventions have shown promising results using a behavioral approach and are recommended for supporting clinical and secondary prevention care for coronary artery disease such as after coronary revascularization (eg, percutaneous coronary intervention) [2]. eHealth has also been shown to be a cost-effective solution essential to increase geographical accessibility to secondary prevention programs, particularly as an addition to existing programs or when other offers are not available. eHealth interventions can be targeted within the natural settings where patients receive access to resources at their discretion [2]. However, patient-related barriers, specifically low health literacy and socioeconomic status, remain obstacles to the large-scale deployment of eHealth in cardiology [1]. Furthermore, patients with low eHealth literacy have lower odds of using eHealth sources to communicate with health care professionals and gain access to health information [3]. Understanding the varying eHealth literacy of patients—defined as the ability to seek, find, understand, and appraise health information from electronic sources and apply the knowledge gained to address or solve a health problem [4]—is thereby pivotal when developing and implementing eHealth resources. Assessing eHealth literacy to identify skill gaps makes it possible to better assist those with low comfort levels in taking advantage of the potential benefit that eHealth can offer, and can empower patients to fully participate in health-related decision making [4]. To assess these benefits, the identification and validation of patient reported outcome measures (PROMs) assessing patients’ perceived eHealth literacy skills are therefore crucial to developing efficient patient-centered eHealth information strategies in the future [5].

To date, there has been limited evidence on PROMs that are most appropriate for assessing eHealth literacy. Systematic reviews have reported that the eHealth literacy scale (eHEALS) was the only PROM used to measure eHealth literacy in more than one study [6,7]. The eHEALS assesses patients’ perceived eHealth literacy skills based on the eHealth literacy Lily model, which combines six literacy types [4,8]. The categories traditional, media, and information literacy are analytic components that involve skills applicable to a broad range of information sources, whereas the scientific, computer, and health literacy categories are context-specific that rely on more situation-specific skills. Combined, these six literacy types form the foundational skills required to fully optimize patients’ experiences with eHealth. The underlying theories of the eHEALS are based in part on self-efficacy theory and social cognitive theory. These two theoretical frameworks promote competencies and confidence as precursors to behavior change and skill development [4,8]. More specifically, the eHEALS is based on the premise that the core literacies in the Lily model are not static but rather process-oriented skills that evolve over time as new technologies are introduced and the personal, social, and environmental contexts change [8]. In this way, the Lily model is clearly related to social cognitive theory, as it is based on a model of causation where behavior, environmental influences, and personal factors all interact and influence each other [9]. This means that eHealth literacy is influenced by a patient’s presenting health issues, educational background, health status at the time of the eHealth encounter, motivation for seeking the information, and the technologies used [4,8].

The eHEALS has been adapted to different languages in Asia [5,10-13] and Europe [14-18]. Furthermore, the psychometrics properties have been evaluated in different populations such as in students [4,12,15,19,20], adults [9,11,16-18,21], and patients with chronic diseases [5,14,22,23], as well as in different cultures in Australia [9,24] and North America [9,17,20-22]. The internal consistency reliability coefficient was shown to be acceptable (ranging from .80 to .90) in the majority of the linguistic versions of the eHEALS [4,11-13,15-19,23], indicating a reliable scale. According to construct validity, the majority of the studies supported a 1-factor model [5,10,12-14,16,22,23,25] recommended by the original scale [4], whereas a few other studies have recommended a 2-factor [11,15,18] or 3-factor [9,21,24] model. However, all of these studies varied contextually when evaluating the dimensions of the eHEALS construct. To our knowledge, no validated version of the eHEALS from the Nordic-Baltic countries has been published to date, and there is limited evidence on its use in patients in the acute coronary care setting such as after percutaneous coronary intervention.

Therefore, in this current study, hypotheses were tested and evaluated against existing knowledge. For instance, a lower eHEALS score has been demonstrated among people with chronic illnesses [3], and differences in eHEALS scores according to age and education have been reported [5,14]. Significant relationships between the eHEALS score and physical and mental health composites among patients with heart failure have been described [23]. An association was also reported between eHealth literacy and health literacy on patients’ perceptions of the usefulness of eHealth in a population with moderate-to-high cardiovascular risk [26], whereas there was a weak-to-moderate correlation between the eHEALS score and health-related internet use among patients with rheumatic disease [14]. This evidence formed the basis for our hypothesis testing according to the COnsensus-based Standards for the selection of health status Measurement INstruments (COSMIN) criteria for validation [27] on the relationship between eHEALS scores and demographic information, health-related internet use, health literacy, and health status in this study (Table 1).

**Table 1:** Hypotheses regarding the relationship between eHEALS^a^ scores and demographic information, health-related internet use, health literacy, and health status based on previous evidence.

Variables		Evidence (relationship with eHEALS)^a^	CONCARD-PCI hypothesis	Analysis
**Demographic information**				
	Age	Weak [14,16,18] or significant [5]	Weak to moderate relationship	Pearson correlation
	Gender	Weak [5,16,18]	Weak relationship	*t* test
	Education	Weak [14,16,18] or significant [5]	Weak relationship	ANOVA^b^
	Employment	Weak [5]	Weak relationship	ANOVA
**Health-related internet use**				
	Used the internet to find information about health	Weak [14], moderate [18], and significant [5]	Moderate relationship	*t* test
	Patient’s interest in using the internet for health information in general (frequency of information-seeking)	Significant [5,16]	Moderate relationship	Spearman correlation
**Health literacy**				
	Ability to find good health information	Moderate [11]	Moderate relationship	Pearson correlation
	Appraisal of health information	Positive [26]	Moderate relationship	Pearson correlation
Health status based on RAND-12^c^ (mental and physical component)		Weak [5] or significant [16,23]	Moderate relationship	Pearson correlation

^a^eHEALS: Electronic health literacy scale.

^b^ANOVA: analysis of variance.

^c^RAND-12: 12-item short-form health survey.

Thus, the aim of this study was to translate and adapt the eHEALS to conditions in Norway, and to determine its psychometric properties. More specifically, we set out to determine the reliability (internal consistency, test-retest) and construct validity (structural validity, hypotheses testing, and cross-cultural validity) of the eHEALS in a self-report format administered to patients after percutaneous coronary intervention.

## Methods

### Design

This validation study used a cross-sectional design and was part of a larger prospective multicenter cohort study, CONCARD^PCI^, which seeks to identify bottlenecks and hurdles in the patient journey, and to suggest the optimal timing of services and alignment with preferences for patients with coronary artery disease undergoing percutaneous coronary intervention [28]. The study adheres to the COSMIN taxonomy of relationships of measurement properties for reliability and construct validity throughout the validation process. The COSMIN taxonomy was developed with the aim to improve the selection of health measurement instruments. It comprises three domains (reliability, validity, and responsiveness), which contain the measurement properties [27]. The COSMIN taxonomy has been widely used for the selection of health measurement instruments for observational studies. To ensure appropriate reporting, the validation study was also performed in accordance with the Strengthening the Reporting of Observational Studies in Epidemiology (STROBE) statements, which constitute an established checklist of items that should be addressed in articles reporting within the three main study designs of analytical epidemiology: cohort, case-control, and cross-sectional studies [29].

### Procedure and Participants

**Figure 1:**
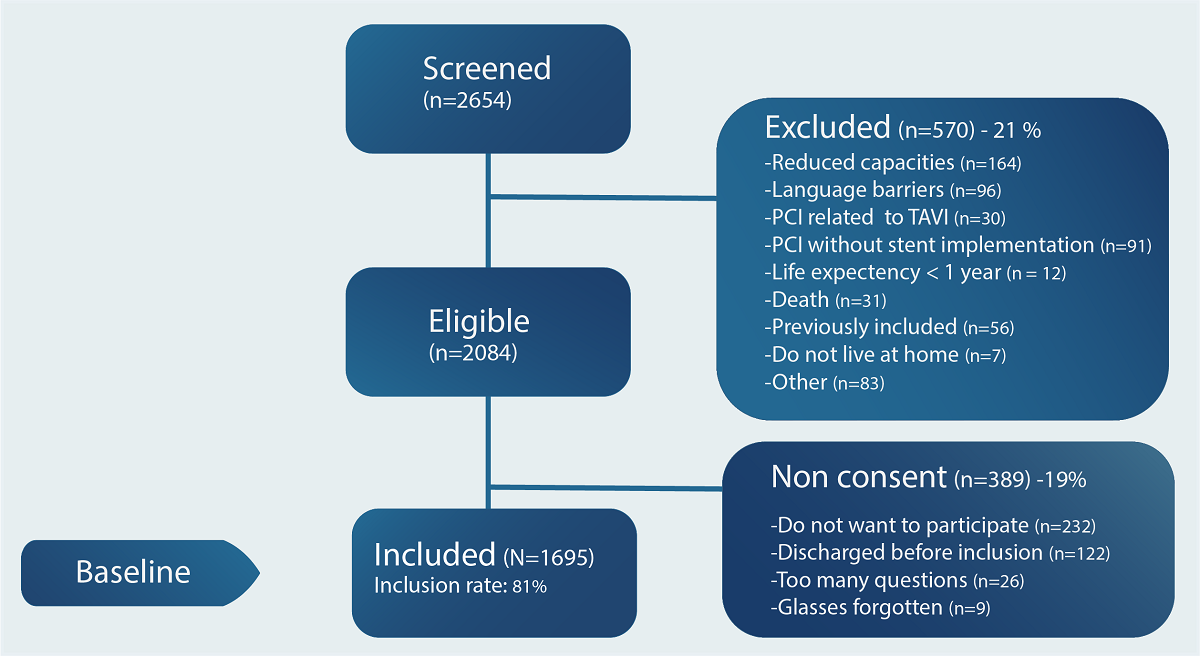
Flow chart of the inclusion process. PCI: percutaneous coronary intervention; TAVI: transcatheter aortic valve implantation.

The study included 1695 patients at index admission for percutaneous coronary intervention at three large Norwegian university hospitals from June 12, 2017 through December 2018 (Figure 1). These three Norwegian university hospitals were selected based on the presence of a committed research team, including CONCARD^PCI^ study nurses and a local principal investigator, and prior research experience, including research infrastructure, geographic location, and size. The percutaneous coronary intervention centers perform between 900 and 2000 (mean 1531) procedures annually, have 482 to 1400 beds (mean 860), and are referral centers for coronary angiography and percutaneous coronary intervention for a total of 17 local hospitals.

Inclusion criteria were patients undergoing percutaneous coronary intervention, ≥18 years of age, living at home at the time of inclusion, and having access to electronic equipment with internet access at the time of inclusion. Exclusion criteria were the inability to speak Norwegian or inability to fill out the self-reported questionnaire due to reduced capacity. To prevent a substantial difference in follow-up time or participants responding based on different assumptions, the patients who were likely to die within less than 1 year were excluded from the study. Institutionalized patients, who may be less likely to have follow up by a primary health care provider or to use the internet to find health information, were also excluded. Similarly, patients undergoing percutaneous coronary intervention without stent implantation and patients undergoing percutaneous coronary intervention related to transcatheter aortic valve implantation or MitralClip often have other indications for the examination or treatment including other follow-up routines and were therefore also excluded from this study.

Self-reports relating to eHealth literacy, health-related internet use, health literacy, health status, as well as demographic information and clinical data identified through the Norwegian Registry for Invasive Cardiology and patient medical records were obtained before discharge from hospital after percutaneous coronary intervention. The self-report was administered using a pencil and paper survey delivered with other PROMs used as part of the CONCARD^PCI^ study. A random subgroup of 100 patients was approached for an eHEALS retest after 2 weeks, 74 (74.0%) of whom completed the retest.

### Translation and Cross-Cultural Adaptation of the eHEALS

A cross-cultural adaptation process was conducted to reach equivalence between the original source and the Norwegian target version of the eHEALS [30]. The translation process was conducted systematically in six steps [30]. Pilot testing was performed with a prefinal version of the eHEALS including 150 patients before being employed in the main cohort study. A summary of the overall translation procedure is described in Textbox 1. The research team encountered some difficulties in translating all of the words and phrases in the original English version of the eHEALS into Norwegian. The original eHEALS questions related to “health resources” were translated as “sources of health information” after approval from the developer of the eHEALS. The patient representatives who participated in the cognitive interviews reported that they clearly understood the items and response options and did not provide any suggestion for additional changes to the prefinal version of the instrument.

**Steps for translation and cross-cultural adaptation of the eHealth literacy scale (eHEALS) into Norwegian.**

- Step 1: Forward translation
  - Two forward translations of the English eHEALS were made by two bilingual translators for whom the target language (Norwegian) was their mother tongue.
  - The translators worked independently, and wrote a report (TL1 and TL2) that identified challenging phrases and described their rationale for final translation choices. An example of a difficult phrase to translate into Norwegian was “health resources.”
  - The two translations were compared and discrepancies were identified.

- Step 2: Synthesis
  - The research team synthesized the reports (TL1 and TL2) into one consensus version (TL3) and described how they resolved discrepancies.

- Step 3: Back translation
  - Two individuals who had a good understanding of English and also spoke Norwegian fluently independently translated TL3 back into English (TL4 and TL5). Neither of the translators who spoke English as their native language was aware of the original version of the eHEALS.

- Step 4: Synthesis and back translation
  - The research team agreed on the modified Norwegian version of the eHEALS (TL6).
  - The research team discussed the timing of administration and meaning of certain words and sentences, and the Likert-type scale.

- Step 5: Instrument pilot testing
  - The prefinal version (TL6) was discussed with patient representatives and piloted before being employed in the large-scale cohort study.
  - A cognitive interview was conducted to test the feasibility and understanding of the items. The patients were asked to read the questionnaire items as well as the instructions.

- Step 6: Revised instrument
  - The researchers evaluated the adapted eHEALS questionnaire (TL6) and all necessary changes were made.
  - Patients who answered that they did not have access to electronic equipment with internet access in the pilot found it challenging to answer the eHEALS items. Therefore, the research team decided that the eHEALS items had low relevance and released these patients from answering the questionnaire.

### Study Instruments and Measures

#### Characteristics of the Study Population

Demographic information collected included age, gender, civil status, smoking status, education level (secondary school, trade school, high school, college/university less than 4 years, college/university 4 years or more), and employment status (working, retired, or other, including sick leave, disability pension, seeking employment). Clinical data included medical history (peripheral vascular disease, stroke, myocardial infarction, diabetes, previous percutaneous coronary intervention, previous coronary artery bypass grafting, previous other heart surgery) and indication for percutaneous coronary intervention (stable angina pectoris, unstable angina pectoris, nonST-segment elevation myocardial infarction [NSTEMI] or ST-segment elevation myocardial infarction [STEMI]).

#### eHealth Literacy Scale

The original English eHEALS comprises eight items and assesses patients’ own perception of their knowledge, comfort, and perceived skills at finding, evaluating, and applying eHealth information [4]. The questionnaire contains two supplementary items to use alongside the eHEALS to better understand patients’ interest in using the internet for health information in general. These items are not a formal part of the eHEALS and are not included in the total score. The original English questionnaire showed high levels of internal consistency (Cronbach α=.88) and modest test-retest reliability [4]. The eHEALS items were used to calculate a mean score using the half rule and were linearly transformed to a 0-100 scale, with higher scores indicating better eHealth literacy. To be able to compare the mean eHEALS score with those reported in other studies, the scale was linearly converted to an 8-40 scale, computed as 8 + (scale from 0 to 100) × (40 – 8)/100.

#### Health-Related Internet Use

To assess patients’ health-related internet use, the following two supplementary items in the eHEALS were used: 1. How useful do you feel the internet is in helping you in making decisions about your health? 2. How important is it for you to be able to access health resources on the internet? [4]. Another question was also developed specifically for this study with “yes” or “no” as the response options: Have you used the internet to find information about health?

#### Health Literacy Questionnaire

The health literacy questions were selected from the Health Literacy Questionnaire (HLQ), which assesses nine separate domains of health literacy. In this study, two domains reflecting skills to use resources and critical evaluation were used: HLQ domain 5 (appraisal of health information, 5 items) and HLQ domain 8 (ability to find good information, 5 items). The first domain had a 4-point response option scale (strongly disagree to strongly agree) and the second domain had a 5-point response option scale (ranging from cannot achieve or always difficult to always easy). The items in the appraisal of the health information domain were used to calculate a total mean score ranging between 1 and 4, and the items in the ability to find good information domain were used to calculate a total mean score between 1 and 5. A low HLQ score indicates that the respondent has difficulties within the domain, and a high score indicates greater health literacy ability. In the event of more than two missing items, the domain score was regarded as missing [31]. The HLQ shows sufficient psychometric properties [31,32], and the Norwegian version of the HLQ has been translated and validated [33].

#### Health Status Questionnaire

The 12-item short-form survey RAND-12 [34] was used to assess overall generic health status through 12 items covering eight domains: physical functioning (2 items), bodily pain (1 item), physical role functioning (2 items), general health (1 item), vitality (1 item), social functioning (1 item), emotional role functioning (2 items), and mental health (2 items), summarized into physical and mental health composite scores. The RAND-12 questionnaire has been validated in European populations and shows sufficient properties [35].

### Expected Relationships and Subgroup Means

The hypotheses testing (convergent validity, known-groups validity, and divergent validity) regarding the relationship between eHEALS scores and demographic information, health-related internet use, health literacy, and health status was formulated a priori. The hypotheses are based on evidence from previous studies on eHEALS as summarized in Table 1.

### Statistical Analyses

We investigated the psychometric properties of the Norwegian version of the eHEALS by assessing the construct validity of three aspects: structural validity, hypotheses testing, and cross-cultural validity [27]. Descriptive statistics were used to summarize patients’ sociodemographic characteristics, clinical data, health-related internet use, health status, and eHEALS scores. Floor and ceiling effects were estimated. Nonparametric tests were used for ordinal variables and parametric tests were used for comparisons of continuous variables. Continuous variables were characterized by the mean (SD). The missing rates for each item were calculated.

The reliability of the eHEALS was assessed by determining its internal consistency and test-retest reliability. Test-retest reliability was calculated by the intraclass correlation coefficient (ICC). Internal consistency reliability (ie, how well the items on a tool fit together) was calculated using Cronbach α, in which α>.70 was considered to be acceptable [27].

Confirmatory factor analyses (CFAs) were used to validate the extent to which the a priori hypotheses concerning dimensions of the eHEALS construct, based on theory and previous analyses, fit the actual data. CFA was used to explore the model fit of eHEALS as a 1-factor model as recommended by the original scale developer [4], in addition to a 2-factor model (information seeking: items 1-5 and 8; information appraisal: items 6 and 7) proposed by Soellner et al [15] and a 3-factor model (awareness: items 1 and 2; skills: items 3-5; and evaluate: items 6-8) proposed by Sudbury-Riley et al [9]. For the CFAs, the robust weighted least square mean and variance adjusted procedure (WLSMV) was used since the items are ordinal. The model fit was evaluated by various goodness-of-fit measures, including the model Chi square statistic with its degrees of freedom and *P* value, in addition to the root mean square error of approximation (RMSEA) (good fit<0.06) and its associated 95% CI, standardized root mean square residual (SRMR; good fit<0.08), comparative fit index (CFI; good fit>0.95), and Tucker-Lewis index (TLI; good fit>0.95) [27].

The convergent validity and divergent validity between the eHEALS and other constructs were assessed by computing Pearson correlation coefficients (*r*) between continuous variables and Spearman correlation coefficients (ρ) between ordinal variables. Correlation coefficients were interpreted such that 0.3 is considered a weak correlation, 0.3 to 0.6 is considered a moderate correlation, and above 0.6 is considered a strong correlation [27,36]. For known-groups validity (eg, gender, education level, employment status, and use of internet), a *t* test or one-way analysis of variance was used. If there was an indication of significant differences (*P*<.05) between groups, a posthoc analysis test for multiple comparisons that does not assume equal variances (Tamhane T2 statistic) was used to investigate where the differences occurred.

SPSS (IBM Corp. Released 2016, IBM SPSS Statistics for Windows, Version 24.0; Armonk, NY, USA) was used for summary statistics and correlations, and for conducting statistical comparisons. Mplus (Computer software, 1998-2018, version 7) developed by BO Muthén and LK Muthen, was used to perform CFAs.

### Ethical Considerations

The study was approved by the Norwegian Regional Committee for Ethics in Medical Research (REK 2015/57). All patients provided written informed consent, and confidentiality and the right to withdraw from the study were assured. The study conformed with the ethical principles outlined in the Declaration of Helsinki.

## Results

### Characteristics of the Study Population

A total of 1695 patients consented to participate in the study (Figure 1). The mean age of the patients was 66 years, ranging from 30 to 96 years. The majority of patients were men, married/living with partner, and hospitalized for an acute coronary event (unstable angina pectoris, NSTEMI, or STEMI). Of the patients included at index admission for percutaneous coronary intervention, a strong majority reported that they have access to electronic equipment with internet access. Most patients reported that they used the internet to find information about health (Table 2). Overall, 37.27% (499/1339) of the patients stated that the internet was useful for making decisions concerning their health and 41.51% (555/1337) stated that it was important to them that they could access health resources on the internet (Multimedia Appendix 1).

**Table 2:** Demographic and clinical characteristics of patients after percutaneous coronary intervention (N=1695).^a^

Characteristic		Value	N^a^
Age (years), mean (SD)		66 (10)	1695
Gender (male), n (%)		1313 (77.46)	1695
**Civil status, n (%)**			1529
	Married/Living with partner	1173 (76.72)	
	Living alone	356 (23.28)	
**Smoking status, n (%)**			1561
	Current smoker	372 (23.83)	
	Previous smoker (>1 month)	713 (45.68)	
	Never smoked	476 (30.49)	
**Education level attained, n (%)**			1522
	Secondary school	331 (21.75)	
	Trade school	543 (35.68)	
	High school	156 (10.25)	
	College/university (<4 years)	269 (17.67)	
	College/university (≥4 years)	223 (14.65)	
**Employed, n (%)**			1544
	Working	559 (36.20)	
	Retired	771 (49.94)	
	Other (sick leave, disability pension, seeking employment)	214 (13.86)	
**Medical history, n (%)**			1685
	Peripheral vascular disease	129 (7.66)	
	Stroke	72 (4.27)	
	Myocardial infarction	346 (20.53)	
	Diabetes	314 (18.63)	
	Previous PCI^b^	426 (25.28)	
	Previous CABG^c^	180 (10.68)	
	Previous other heart surgery	19 (1.13)	
**Indication for PCI, n (%)**			1695
	SAP^d^	473 (27.91)	
	UAP^e^	266 (15.69)	
	NSTEMI^f^	522 (30.80)	
	STEMI^g^	346 (20.41)	
	Other	88 (5.19)	
Access to electronic equipment with internet access, n (%)		1402 (93.66)	1497
Used the internet to find information about health, n (%)		980 (66.08)	1483

^a^Number of observations for each characteristic may not total 1695 because of missing data.

^b^PCI: percutaneous coronary intervention.

^c^CABG: coronary artery bypass grafting.

^d^SAP: stable angina pectoris.

^e^UAP: unstable angina pectoris.

^f^NSTEMI: nonST-segment elevation myocardial infarction.

^g^STEMI: ST-segment elevation myocardial infarction.

### Psychometric Analyses

#### General Properties

The mean eHEALS score was 25.66 (SD 6.23). The highest mean of the eHEALS items was 3.40 and the lowest mean was 2.92. Among all respondents, 80% were most likely to select one (41%) or two (39%) response options across all items, with 34%-51% responding “undecided” and 22%-47% responding “agree” (Multimedia Appendix 1). In total, 45 (3%) maximum possible scores and 27 (2%) minimum possible scores were identified, indicating limited ceiling and floor effects. The total mean eHEALS score for the retest was 53.52 (SD 19.79), with a floor of 5.6% (n=4) and ceiling of 1.4% (n=1).

#### Reliability

Cronbach α for the eHEALS was >.99 (Table 3). The corresponding Cronbach α for the 2-week retest was .94. The ICC for the eHEALS was 0.605 (95% CI 0.419-0.743, *P*<.001), indicating moderate stability over time.

**Table 3:** Mean (SD) scores and Cronbach α values of the eHEALS^a^, HLQ^b^, and RAND-12^c^ of patients after percutaneous coronary intervention (N=1659).

Item			Mean (SD)	Cronbach α
eHEALS^a^			25.66 (6.23)	>.999
**HLQ^b^**				
		HLQ 5^e^	2.43 (0.66)	.844
		HLQ 8^d^	3.22 (0.73)	.875
**RAND-12^e^**				
		PCS^f^12	43.93 (10.88)	N/A^g^
		MCS^h^12	46.48 (11.14)	N/A

^a^eHEALS: eHealth literacy scale.

^b^HLQ: health literacy questionnaire.

^c^HLQ 5:Appraisal of health information.

^d^HLQ 8: Ability to find good health information.

^e^RAND-12: 12-item short-form health survey.

^f^PCS: physical composite score.

^g^N/A: not applicable; since PCS12 and MCS12 of RAND-12 are not computed as means or sum scores, there is no Cronbach α.

^h^MCS: mental health composite score.

#### Structural Validity

The strong standardized factor loadings for the 1-factor model, ranging from 0.79 to 0.93, indicated promising item properties. The Chi square test of model fit (*P*<.001), SRMR, CFI, and TLI indices suggested a good fit. However, the high RMSEA value suggested a poor structural fit of the eHEALS in the 1-factor model.

For the 2-factor model, standard factor loadings ranged from 0.80 to 0.93. Similar to the 1-factor model, this model suggested a good fit based on the SRMR, CFI, and TLI, and a poor fit for the RMSEA (Table 4). Furthermore, examination of the modification indices through pairing items 5 and 8 to the second (appraisal) factor did not suggest an appreciable improvement in fit for the RMSEA, although it was slightly reduced (0.176, 90% CI 0.165-0.187).

**Figure 2:**
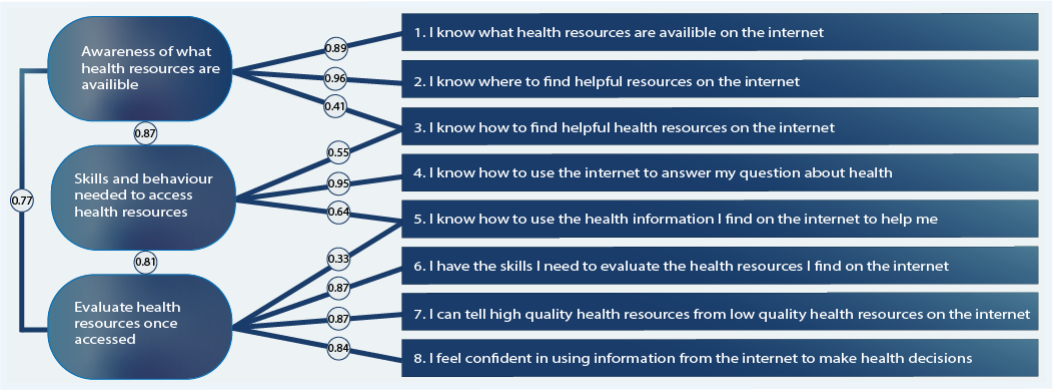
Electronic health literacy scale (eHEALS) 3-factor model proposed by Sudbury-Riley et al [9] with modification for items 1 and 5.

Standard factor loadings in the 3-factor model ranged from 0.84 to 0.97. Similar to the 1- and 2-factor models, the CFA supported a good fit for the three indices SRMR, CFI, and TLI, whereas the RMSEAs remained high (Table 4). Examination of the modification shown in the output file conducted by pairing item 3 in the first (awareness) and second (skills) factor, and item 5 in the second (skills) and third factor (evaluate) suggested an improvement in the model fit (SRMR=0.008, CFI=0.999, TLI=0.997, RMSEA=0.057; 90% CI 0.045-0.070). The standard factor loadings and correlations for the modifications are presented in Figure 2.

**Table 4:** Goodness-of-fit indices of the eHEALS^a^ 1-, 2-, and 3-factor structure model.

Model	Chi square (df)	RMSEA^b^ (90% CI)	SRMR^c^	CFI^d^	TLI^e^
Model 1^f^	1649.256 (20)	0.247 (0.237-0.257)	0.045	0.966	0.952
Model 2^g^	1482.130 (19)	0.240 (0.230-0.251)	0.040	0.969	0.955
Model 3^h^	510.925 (17)	0.148 (0.137-0.159)	0.019	0.990	0.983

^a^eHEALS: eHealth literacy scale.

^b^RMSEA: root mean square error of approximation.

^c^SRMR: standardized root mean square residual.

^d^CFI: comparative fit index.

^e^TLI: Tucker-Lewis index.

^f^1-factor model: Factor 1:1-8 [4].

^g^2-factor model: Factor 1: 1-5, 8; Factor 2: 6, 7 [15].

^h^3-factor model: Factor 1: 1, 2; Factor 2: 3-5; Factor 3: 6-8 [9].

#### Convergent and Discriminant Validity

##### Demographic Information

**Figure 3:**
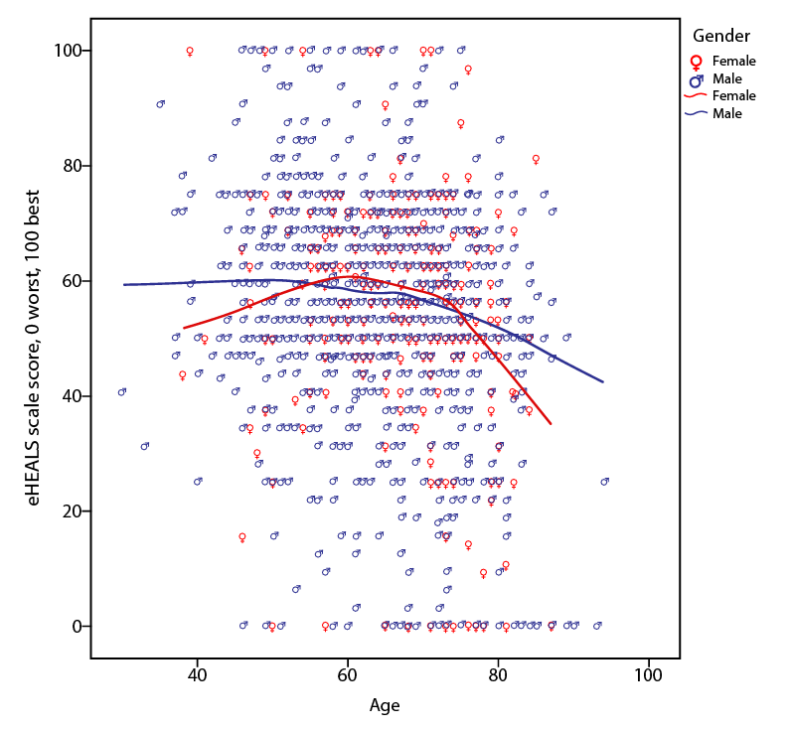
Association between electronic health literacy scale (eHEALS) scores, gender, and age. The scale was linearly transformed to a 0-100 scale. The scale was linearly converted to an 8-40 scale (scale from 8 to 40 computed as 8 + [scale from 0 to 100] × [40 – 8]/100). The eHEALS scale in the figure is: 0=8, 20=14.4, 40=20.8, 60=27.2, 80=33.6, 100=40.

Pearson correlation analysis showed a weak negative correlation between the eHEALS score and age. An independent-sample *t* test did not indicate substantial differences in the eHEALS score between men (mean 25.64, SD 6.15) and women (mean 25.47, SD 6.60) (t_1313_=–0.526, *P*=.60) (Table 5 and Figure 3).

As shown in Table 5, the between-group analysis of variance showed a difference according to educational groups in the eHEALS score. A posthoc test indicated that patients with 4 or more years of college/university education scored higher on the eHEALS compared to those with secondary school (mean difference=4.61, *P*<.001), trade school (mean difference=3.23, *P*<.001), and high school (mean difference=2.24, *P*=.002) education. Patients with less than 4 years of college/university education scored higher on the eHEALS than those with secondary (mean difference=3.39, *P*<.001) and trade school (mean difference=2.00, *P*<.001) education. Patients with high school education had higher eHEALS scores than those with secondary school education (mean difference=2.37, *P*<.001).

The between-groups analysis of variance also indicated a difference according to employment groups and eHEALS scores (Table 5). The posthoc test indicated a higher eHEALS score for patients who were employed compared to those who were retired (mean difference=2.31, *P*<.001). Similarly, the “other” patients group showed a higher eHEALS score compared to that of the retired patients (mean difference=1.28, *P*=.05).

##### Health-Related Internet Use

An independent sample *t* test showed a higher eHEALS score for patients who reported that they used the internet to find information about health (mean 27.45, SD 5.10) than that of patients who did not (mean 21.27, SD 6.31) (95% CI –21.40 to –17.21, *P*<.001). Spearman correlation analysis revealed a moderate positive correlation between the eHEALS score and patients’ perceived usefulness and importance of using the internet to find health information (Table 5).

##### Health Literacy

Pearson correlation analysis revealed a moderate positive correlation between the eHEALS score and the HLQ scale for appraisal of health information and the HLQ scale for ability to find good information (Table 5).

##### Health Status

Pearson correlation analysis revealed a weak positive correlation between the eHEALS score and self-reported health assessed with RAND-12 (Table 5).

**Table 5:** Group statistics and correlations between eHEALS^a^ score, patients’ demographic, and other instruments.

Variable		Statistic	*P* value
**Demographic information**			
	Age, Pearson correlation coefficient	–0.206	<.001
	Gender, 95% CI	–3.38-1.95	.60
	Education, *F*_4,1280_ (ANOVA^b^)	21.085	<.001
	Employment, *F*_2,1296_ (ANOVA)	19.615	<.001
**Health-related internet use**			
	Use of internet, 95% CI	–21.40 to –17.21	<.001
	eHEALS supp. 1^c^, Spearman correlation coefficient	0.587	<.001
	eHEALS supp. 2^d^, Spearman correlation coefficient	0.574	<.001
**Health literacy**			
	HLQ^e^ 5^f^, Pearson correlation coefficient	0.380	<.001
	HLQ 8^g^, Pearson correlation coefficient	0.561 (<.001)	<.001
**Health status (RAND-12^h^)**			
	Mental component, Pearson correlation coefficient	0.116	<.001
	Physical component, Pearson correlation coefficient	0.112	<.001

^a^eHEALS; eHealth literacy scale.

^b^ANOVA: analysis of variance.

^c^eHEALS supp.1: How useful do you feel the internet is in helping you in making decisions about your health?

^d^eHEALS supp.2: How important is it for you to be able to access health resources on the internet?

^e^HLQ: health literacy questionnaire.

^f^HLQ domain 5: appraisal of health information.

^g^HLQ domain 8: ability to find good information.

^h^RAND-12: 12-item short-form health survey.

## Discussion

### Principal Findings

To our knowledge, this is the first study to determine the psychometric properties of the eHEALS in patients after percutaneous coronary intervention. The Norwegian translation of the eHEALS appears to have acceptable construct validity. However, the high internal item consistency and the high RMSEA suggest that the fit of the data to the hypothesized models based on existing knowledge is not entirely adequate to fully capture the construct validity in this setting.

The structural validity was confirmed by three (SRMR, CFI, and TLI) out of four goodness-of-fit indices, indicating an adequate fit of the three hypothesized models. The RMSEA was lower in the 3-factor model [9] than in the 1-factor [4] and 2-factor [15] models. After two modifications of the 3-factor model, all four goodness-of-fit indices indicated a good fit. These results suggest that the eHEALS is a multidimensional construct that, as proposed by other studies, is a better fit for the 2-factor model [11,15,18] and 3-factor model [9,21,22,24]. However, in line with the current study, several previous studies conducting CFA showed an RMSEA value above the cut-off criteria for the 1-factor model [9,11,22], 2-factor model [15,18,22], and 3-factor model [9,24]. The high RMSEA indicated a poor fit, suggesting that complexity exists in all three models, but the 3-factor model was found to have acceptable fit after a low number of reasonable modifications. The differences in model fit among the three CFA models suggest that it is possible to distinguish between different conceptualizations even with high redundancy.

A high proportion of patients were most likely to select the response “undecided” or “agree” across all items, suggesting that most of the patients either considered themselves as neutral (neither disagree or agree) or relatively confident about their knowledge, comfort, and perceived skills at finding, evaluating, and applying eHealth information. Although there is inconclusive evidence about the number of categories in a response scale and whether the neutral category has an impact on measurement quality [37], it has been recommended that future research should explore which response options are most appropriate to include in the eHEALS to obtain a more precise measure of eHealth literacy [22]. One explanation for the high proportion of respondents most likely to select these two response options could be that the patients experienced difficulties filling out the questionnaire in the context of an acute coronary event. Furthermore, patients with acute coronary syndrome may be more prone to survey response bias in such a manner that they select a neutral option irrespective of their actual attitude or behavior. This suggests that the scale may work differently for patients with chronic diseases than for patients in acute settings. This finding also underpins that eHealth literacy is a process-oriented skill that evolves over time as new technologies are introduced and the personal, social, and environmental contexts change [8]. Similarly, it is also possible that the very high estimate of internal consistency is attributed to the patients’ difficulties in differentiating between the meanings of the items in an acute coronary setting. A Cronbach α value above .90 has also been reported in other studies [5,10,14]. However, the very high Cronbach α in this study may indicate that there is a potential redundancy of items, wherein patients immediately after percutaneous coronary intervention may perceive that the same items are essentially rephrased in several different ways. The need for further research investigating item interpretation across populations has been suggested [14,24].

The current study indicates adequate discriminant validity of the eHEALS, and most of the demographic information and other instruments confirmed the hypotheses defined a priori. As confirmed in previous psychometric studies within general adult European populations [14,16,18], the measurement properties of the eHEALS were affected by age and gender to a lesser extent. However, the Chinese version showed a difference in eHEALS scores according to age among chronic disease patients [5]. In addition, the latter study showed that the eHEALS score was affected by education levels [5], whereas other studies reported a weak correlation with education in general adult European populations [14,18]. The results of previous studies and those of the current study diverge in this respect, indicating insufficient evidence to link perceived eHealth literacy with education. However, these findings may also be related to the fact that different methods were used to categorize and analyze education levels, and therefore should be interpreted with caution.

As the promotion of eHealth literacy takes place within a larger context, the original scale developer recommended involving other groups engaged in the literacy sectors in the work on validating the eHEALS [4]. Additionally, to address the patients’ eHealth literacy level, patient integration in the evaluation is of great importance, specifically in accordance with PROMs such as health-related internet use, health literacy, and health status. A moderate correlation between the eHEALS score and patients’ interest in using the internet for health information in general has been suggested, which strengthens the discriminant validity [16,18]. This provides a direction for skills, motivation, and interest that is applicable to a broad range of information sources, irrespective of the topic or context, in line with the analytic components of the Lily model [8]. Furthermore, according to the context-specific nature of eHealth literacy skills [8], a moderate correlation was found between the eHEALS score and health literacy. Consistent with previous studies that showed a relationship between eHealth literacy and health literacy [11,26], the current study indicates that patients with higher eHEALS scores tend to be “information explorers,” able to identify good information and reliable sources of information, and to resolve conflicting information by themselves or with the help of others [31]. This relationship suggests that the construct validation in the scale is adequate. However, this study also showed a weak correlation between the eHEALS score and health status. This differs from other studies [16,23], suggesting modest divergent validity of the eHEALS in terms of its relationship with health status.

### Strengths and Limitations

The current study has several methodological strengths and limitations that should be addressed. The stringent linguistic, cultural, and measurement adaptation procedures are likely to have contributed to strengthening the conduct of the study. However, the Norwegian eHEALS showed mixed psychometric performance, which is likely due to the context of an acute coronary event. This indicates that hospitalization can affect the response to this type of PROM. Another key strength of the study is the large sample size, which allowed us to investigate the correlations between eHEALS scores, other PROMs, and subgroups. However, the analysis of the translated eHEALS was determined to be specific to patients who underwent percutaneous coronary intervention and cannot be generalized to other scenarios. There is therefore a need to determine the psychometric properties of the eHEALS in a more diverse population and in other settings to provide empirical evidence of the generalizability of the Norwegian eHEALS. Finally, the study only determined the administration of the eHEALS in self-reported written format (paper and pencil) in a hospital acute care setting. Further work should explore other modes of administration, including online administration developed for eHealth sources such as tablets, smartphones, and email.

### Conclusion

This study provides new information on the psychometric properties of the eHEALS for patients after percutaneous coronary intervention, suggesting that the eHEALS is a multidimensional construct. Nevertheless, the RMSEA is not entirely adequate to fully capture the construct validity based on existing knowledge, and further factorial validation studies are needed. The internal item consistency was very high, indicating a redundancy of items. There is nonetheless a need for more research on the psychometric properties of the eHEALS. Moreover, use of the eHEALS in this study identified areas of eHealth literacy that are important for the further development of eHealth as a source of health information.

## Appendix

Multimedia Appendix 1 Response frequencies (%) and mean (SD) for all the eHEALS items, including supplementary items (N=1695).

## Abbreviations

CFA: confirmatory factor analysis
CFI: comparative fit index
COSMIN: Consensus-based standards for the selection of health status measurement instruments
eHEALS: electronic health literacy scale
eHealth: electronic health
HLQ: health literacy questionnaire
ICC: intraclass correlation coefficient
NSTEMI: nonST-segment elevation myocardial infarction
PROM: patient reported outcome measure
RAND-12: 12-item short-form health survey
RMSEA: root mean square error of approximation
SRMR: standardized root mean square residual
STEMI: ST-segment elevation myocardial infarction
STROBE: Strengthening the Reporting of Observational Studies in Epidemiology
TLI: Tucker-Lewis index
