# Supplementary material for: Psychometric Properties of the Norwegian Version of the Electronic Health Literacy Scale (eHEALS) Among Patients After Percutaneous Coronary Intervention: Cross-Sectional Validation Study
Source: J Med Internet Res. 2020 Jul 28;22(7):e17312. doi: 10.2196/17312 (PMC7420812; doi:10.2196/17312)
Supplement: Multimedia Appendix 1 [file jmir_v22i7e17312_app1.docx]

**Multimedia Appendix 1:** Response frequencies (%) and mean (SD) for all the eHEALS items, including supplementary items (N=1695).

| Items | n (%) | n (%) | n (%) | n (%) | n (%) | Mean(SD) | N |
| --- | --- | --- | --- | --- | --- | --- | --- |
|  | Not useful at all | Not useful | Unsure | Useful | Very useful |  |  |
| Supp.1. How useful do you feel the internet is in helping you in making decisions about your health? | 121 (9.04) | 121 (9.04) | 489 (36.52) | 499 (37.27) | 109 (8.14) | 3.26 (1.04) | 1339 |
|  | Not important at all | Not important | Unsure | Important | Very important |  |  |
| Supp.2. How important is it for you to be able to access health resources on the internet? | 94 (7.03) | 147 (10.99) | 340 (25.43) | 555 (41.51) | 201 (15.03) | 3.47 (1.09) | 1337 |
|  | Strongly disagree | Disagree | Undecided | Agree | Strongly agree |  |  |
| 1. I know what health resources are available on the internet. | 76 (5.47) | 141 (10.15) | 594 (42.76) | 448 (32.25) | 59 (4.25) | 3.21 (0.90) | 1389 |
| 1. I know where to find helpful health resources on the internet. | 69 (5.01) | 120 (8.71) | 510 (37.01) | 547 (19.70) | 61 (4.43) | 3.31 (0.90) | 1378 |
| 1. I know how to find helpful health resources on the internet. | 72 (5.24) | 98 (7.13) | 448 (32.61) | 612 (44.54) | 73 (5.31) | 3.40 (0.91) | 1374 |
| 1. I know how to use the internet to answer my questions about health. | 69 (4.99) | 111 (8.03) | 463 (33.48) | 578 (41.79) | 91 (6.58) | 3.39 (0.93) | 1383 |
| 1. I know how to use the health information I find on the internet to help me. | 70 (5.08) | 116 (8.41) | 603 (43.73) | 450 (32.63) | 69 (5.00) | 3.25 (0.89) | 1379 |
| 1. I have the skills I need to evaluate the health resources I find on the internet. | 105 (7.63) | 189 (13.73) | 556 (40.38) | 382 (27.74) | 74 (5.37) | 3.10 (0.99) | 1377 |
| 1. I can tell high quality health resources from low quality health resources on the internet. | 98 (7.11) | 149 (10.81) | 666 (48.33) | 328 (23.80) | 66 (4.79) | 3.09 (0.93) | 1378 |
| 1. I feel confident in using information from the internet to make health decisions. | 140 (10.13) | 208 (15.05) | 627 (45.37) | 290 (20.98) | 46 (3.33) | 2.92 (0.97) | 1382 |

Abbreviation: Supp.; Supplementary. Number of observations for each characteristic may not total 1695 because of missing data.
